# Five-year stroke prognosis. Influence of post-stroke delirium and post-stroke dementia on mortality and disability (Research Study – Part of the PROPOLIS Study)

**DOI:** 10.1007/s10072-023-07129-5

**Published:** 2023-10-18

**Authors:** Jakub Droś, Natalia Segiet, Gabriela Początek, Aleksandra Klimkowicz-Mrowiec

**Affiliations:** 1 Doctoral School in Medical and Health Sciences, Jagiellonian University Medical College, Kraków, Poland; 2 The Doctoral School of the Medical University of Silesia, Katowice, Poland; 3 Department of Internal Medicine and Gerontology, Faculty of Medicine, Jagiellonian University Medical College, Ul. Jakubowskiego 2, 30-688 Kraków, Poland

**Keywords:** Stroke, Mortality, Delirium, Cognition disorders, Dementia

## Abstract

**Introduction:**

With increasing life expectancy and the rising incidence of stroke in young adults, it is important to know the long-term prognosis of this condition. Post-stroke delirium and post-stroke dementia are common complications of stroke that negatively affect prognosis. The purpose of this study was to evaluate five-year mortality from stroke and to assess the influence of post-stroke delirium and post-stroke dementia on mortality and disability over the five-year period.

**Methods:**

Consecutive patients admitted to the stroke unit for acute stroke or transient ischemic attacks were screened for in-hospital delirium. At the three- and twelve-month follow-up, the same patients underwent neurocognitive testing. Diagnoses of in-hospital delirium and dementia after three and twelve months based on DSM-5 criteria. Five years after stroke surviving patients were reevaluated. Outcome assessment included place of stay, current functional status assessed by the modified Rankin Scale (mRS), or death.

**Results:**

At the five-years of follow-up, data were collected from 575 of 750 patients originally included in the study (76.67%). The mortality rate was 51.65%. In-hospital post-stroke delirium and post-stroke dementia diagnosed three and twelve months after stroke were independent risk factors for death and an increase in mRS score of ≥ 1 or ≥ 2 points. There was no significant association with institutionalization rate.

**Conclusions:**

More than half of post-stroke patients die within five years of follow-up. Post-stroke delirium and post-stroke dementia are associated with an increased risk of death and disability.

## Introduction

According to a recent World Health Organization report, stroke remains the second most common cause of death and disability [1, 2]. Stroke significantly increases the risk of mortality compared to the general population at the same age [3]. Giving the increasing life expectancy and the rising incidence of stroke in young adults [4, 5], knowledge of the prognosis many years after stroke is very important.

Among stroke survivors, it has a direct impact on short- and long-term consequences including neuropsychiatric and motor disorders, which are often associated with severe disabilities and functional limitations [6, 7]. Among them, post-stroke delirium and post stroke dementia are very frequent and require special attention [8, 9]. Delirium, a transient state of impaired attention and consciousness, is one of the most common complication in acute hospital admissions, leading to higher rates of post-discharge mortality and institutionalization [10]. Dementia is a disorder of multifactorial causes, overlapping symptoms and heterogenous pathologies resulting in impaired cognitive functioning and behavioral abilities that affect performing activities of daily living [11]. Although the literature on the impact of post-stroke delirium and post-stroke dementia on further prognosis is extensive, the number of studies on patients at very long follow-up is still insufficient.

The purpose of this study was to evaluate five-year mortality after stroke and answer the question whether post-stroke delirium and post-stroke dementia affect mortality and disability in five-year perspective.

## Methods

The present study was part of a large single-center PRospective Observational POLIsh Study on post-stroke delirium (“PROPOLIS”) conducted at Jagiellonian University Medical College in Kraków, Poland. All procedures carried out in this study involving human subjects were in accordance with the ethical standards of the institutional and national research committee and with the 1964 Helsinki declaration and its later amendments. Each participant or his/her guardian gave written consent to participate in the study. The study protocol was approved by the Jagiellonian University Bioethics Committee (1072.6120.177.2021).

Consecutive patients with acute stroke or transient ischemic attack (TIA) admitted to the Stroke Unit of the University Hospital in Kraków meeting the inclusion criteria (patients > 18 years of age, admitted within 48 h of the first stroke symptoms, Polish-speaking) were included in the study. Stroke was defined as a sudden onset of neurological deficit lasting more than 24 h. All patients were treated according to standard protocols of international guidelines. Detailed exclusion criteria were described in the study protocol [12].

During the hospital stay, data were collected on socio-demographic factors (age, gender, education), medical history (comorbidities, medications, infections, laboratory results), and stroke-related factors (type of stroke, severity, stroke symptoms). The Cumulative Illness Rating Scale (CIRS) was used as a general indicator of health status [13]. On admission, information was obtained from a spouse or a caregiver regarding pre-stroke cognitive functioning based on the Polish version of Informant Questionnaire on Cognitive Decline in the Elderly (IQCODE) [14]. Disability prior to hospital admission was assessed by the modified Rankin Scale (mRS) [15].

All patients underwent neuroimaging (computed tomography/magnetic resonance imaging) during hospital admission. The severity of clinical deficit was assessed using the National Institutes of Health Stroke Scale (NIHSS) [16]. In addition, ischemic stroke subtype was evaluated using the Trial of ORG 10172 in Acute Stroke Treatment (TOAST) classification [17]. Data on aphasia, neglect or vision deficits were also collected.

Every day, from admission to the seventh day of hospitalization, patients were screened for delirium using an abbreviated version of the Confusion Assessment Method (bCAM) [18]. For patients with motor aphasia or those who could not communicate for other reasons, the Intensive Care Unit version (CAM-ICU) was used [19]. The diagnosis of delirium followed the criteria in the Diagnostic and Statistical Manual of Mental Disorders, Fifth Edition (DSM-5) [20] and was based on clinical observations and structural assessments by a neurologist/neuropsychologist and ward nurses. All details of the in-hospital assessment of post-stroke delirium have been described elsewhere [21, 22].

All patients discharged from the hospital were scheduled for a follow-up visit three and twelve months after the stroke to undergo a neuropsychological examination. In patients who refused full neuropsychological examination, only Montreal Cognitive Assessment (MoCA) was performed [23]. We used the following MoCA cut-off points for dementia: ≤ 20 at three-month follow-up and ≤ 23 at twelve-month follow-up [24]. Patients who were n0t able to come to the clinic were assessed using the telephone version of MoCA (T-MoCA) [25]. Scores from the T-MoCA were proportionally scaled relative to the maximum score that could be obtained. Katz et al. presented a new method for recalculating T-MoCA scores based on a well‐characterized, demographically diverse cohort of older adults [26]. The cut-off points for dementia in T-MoCA are the same (15 and 17 points, respectively) after applying both the proportional conversion we used previously [21] and the method described by Katz et al. To assess cognitive status in patients who were unavailable for in-person and telephone interviews because of severe impairments, the IQCODE was conducted with a caregiver. Dementia was diagnosed if the mean score was ≥ 4.0 [14, 27]. In a population of Polish stroke patients, a mean score of ≥ 4.0 has 85% sensitivity and 94% specificity as previously described [14]. The final diagnosis of post-stroke dementia based on the cognitive and functional assessment, and information provided by a caregiver. The DSM-5 diagnostic criteria for major neurocognitive disorder [20] were used.

We then contacted stroke survivors again, five years after stroke, and invited them for the follow-up visit and reassessment. Patients who did not attend the appointment were interviewed by telephone. In cases where the patients could not be interviewed, the patient’s caregiver was contacted and interviewed. Outcome assessment included place of stay (home, rehabilitation hospital or long-term institution), current functional status (mRS), or death. Mortality data were collected when the investigator was reliably informed of the participant’s death, usually by a close informant from the participant’s household. A participant was considered to be lost to follow-up if we were unable to contact him or her three times on three different days.

## Statistical analyses

Statistical analyses were performed using Statistica 13.3 software (StatSoft®, Poland). To search for independent risk factors for mortality, univariate logistic regression models including considerable demographic and clinical factors were performed, followed by a multivariate logistic regression model, adjusted for relevant variables at *P*-value < 0.01. To analyze the impact of post-stroke delirium and dementia on further prognosis, its predictive values were calculated for death, increase in mRS score of ≥ 1 or ≥ 2 points, and hospital or institution stay. Each outcome analysis included multivariate logistic regression model adjusted for age, gender, years of education, CIRS score and NIHSS score. Two multivariate logistic regression models were run to assess the impact of post-stroke dementia on prognosis – the second was adjusted also for the incidence of in-hospital delirium. Kaplan–Meier curves were used to represent five-year survival according to prevalence of delirium and dementia. The Gehan-Wilcoxon test was used to compare mortality between the groups. Continuous values were presented as medians with interquartile ranges (IQRs), and predictive values as odds ratios (ORs) with 95% confidence intervals (CIs). *P*-values < 0.05 were considered statistically significant.

## Results

**Fig. 1:**
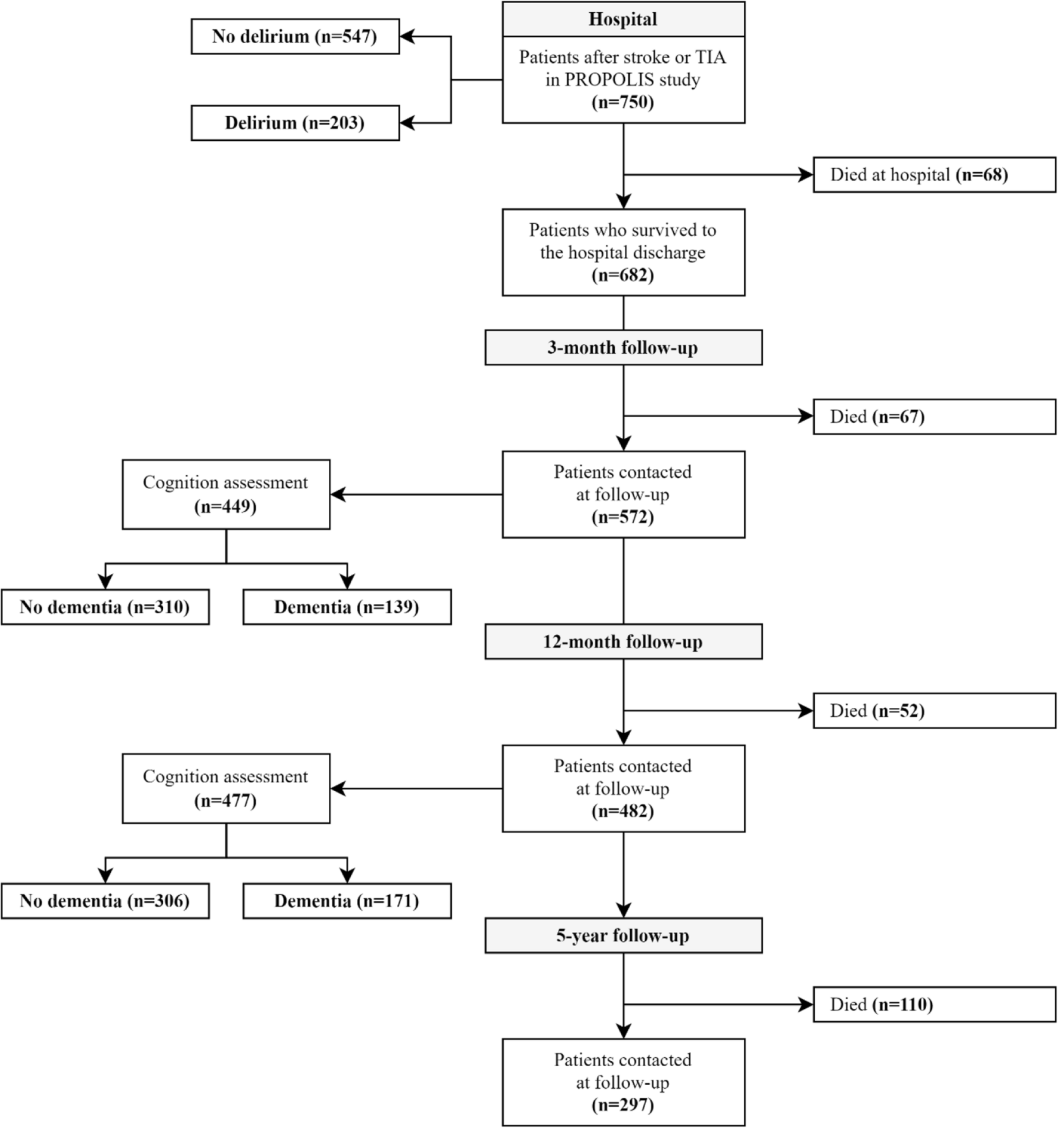
Study flowchart

Out of 750 consecutive stroke patients were initially enrolled in the “PROPOLIS” study (650 with ischemic stroke, 52 with hemorrhagic stroke and 48 with TIA), data from 575 cases (76.67%) were collected at the five-year follow-up (498 with ischemic stroke, 44 with hemorrhagic stroke and 33 with TIA). Study flowchart is presented in Fig. 1.

A total of 152 patients (26.43%) attended the visit in the outpatients clinic, and 126 (21.91%) were contacted by phone and interviewed. Ten of 278 stroke survivors (3.60%) were in a rehabilitation hospital or long-term institution. Remaining 297 participant died within five years after stroke, and the mortality rate was 51.65%. 175 cases (23.33%) were lost to follow-up. Table 1 shows a comparison of socio-demographic, medical and stroke-related factors between included and lost to follow-up cases.

**Table 1:** Comparison of included and lost to follow-up cases

VARIABLE	Data	INCLUDED CASES (*n* = 575)	LOST TO FOLLOW-UP (*n* = 175)	*P*-value
Male gender †	750	266/575 (46.26%)	86/175 (49.14%)	0.504
Age [years] ‡	750	74 (63–82)	71 (64–80)	0.338
BMI [kg/m^2^] ‡	730	26.70 (23.62–29.98)	26.05 (24.22–30.25)	0.879
Length of education [years] ‡	658	11 (9–13)	11 (8–13)	0.069
Medical history				
- hypertension †	747	407/572 (71.15%)	126/175 (72.00%)	0.828
- diabetes †	747	164/572 (28.67%)	39/175 (22.29%)	0.092
- atrial fibrillation †	748	149/573 (26.00%)	31/175 (17.71%)	0.021
- myocardial infraction †	747	81/572 (14.16%)	25/175 (14.29%)	0.967
- PCI or CABG †	747	48/572 (8.39%)	15/175 (8.57%)	0.940
- smoking – ever †	725	244/553 (44.12%)	87/172 (50.58%)	0.138
- previous stroke or TIA †	741	111/567 (19.58%)	35/174 (20.11%)	0.876
CIRS, total score ‡	747	9 (6–13)	9 (5–13)	0.350
Aphasia in hospital †	750	205/575 (35.65%)	50/175 (28.57%)	0.080
Neglect in hospital †	750	73/575 (12.70%)	21/175 (12.00%)	0.807
Vision deficits in hospital †	750	246/575 (42.78%)	52/175 (29.71%)	0.002
Delirium in hospital †	750	166/575 (28.87%)	37/175 (21.14%)	0.040
NIHSS at admission ‡	750	7 (3–15)	4 (2–9)	< 0.001
Pre-hospital mRS ‡	749	0 (0–1)	0 (0–0)	0.023
- pre-hospital mRS = 0 †	749	394/574 (68.64%)	138/175 (78.66%)	0.001
- pre-hospital mRS = 1 †	749	57/574 (9.93%)	15/175 (8.57%)	
- pre-hospital mRS = 2 †	749	34/574 (5.92%)	6/175 (3.43%)	
- pre-hospital mRS = 3 †	749	37/574 (6.45%)	12/175 (6.86%)	
- pre-hospital mRS = 4 †	749	26/574 (4.53%)	4/175 (2.29%)	
- pre-hospital mRS = 5 †	749	26/574 (4.53%)	0/175 (0%)	
Pre-hospital IQCODE ‡	609	78 (78–84)	78 (78–81)	0.551
CRP level in hospital [mg/l] ‡	729	7.38 (2.41–20.88)	6.14 (2.48–16.44)	0.195

^†^ *n* (%) ‡ median (IQR)

Significant predictors of death in the univariate logistic regression analysis are shown in Table 2. The multivariate logistic regression model revealed older age (OR 1.114, 95%CI 1.079–1.151, *p* < 0.001), presence of atrial fibrillation (OR 2.266, 95%CI 1.145–4.487, *p* = 0.019), smoking before stroke (OR 2.817, 95%CI 1.472–5.391, *p* = 0.002), visual deficits in hospital (OR 2.968, 95%CI 1.626–5.416, *p* < 0.001), in-hospital delirium (OR 2.887, 95%CI 1.433–5.813, *p* = 0.003), pre-hospital mRS (OR 1.525, 95%CI 1.172–1.984, *p* = 0.002) and higher in-hospital CRP levels (OR 1.011, 95%CI 1.003–1.020, *p* = 0.006) as independent risk factors of mortality five years after stroke. These results are shown in Table 3.

**Table 2:** Analysis of risk factors of five-year mortality for survivors and non-survivors in the univariate logistic regression model

VARIABLE	Data	SURVIVORS (*n* = 278)	NON-SURVIVORS (*n* = 297)	OR (95%CI)	*P*-value
Male gender †	575	140/278 (50.36%)	126/297 (42.42%)	0.726 (0.523–1.009)	0.057
Age [years] ‡	575	65 (58–73)	81 (73–86)	1.120 (1.098–1.142)	< 0.001
BMI [kg/m^2^] ‡	558	25.93 (23.44–29.90)	27.21 (24.09–29.98)	1.020 (0.985–1.056)	0.274
Length of education [years] ‡	500	12 (10–14)	10 (8–12)	0.875 (0.827–0.926)	< 0.001
Hemorrhagic stroke †	575	18/278 (6.47%)	26/297 (8.75%)	1.386 (0.742–2.588)	0.306
TOAST classification					
- large-artery atherosclerosis †	497	26/236 (11.02%)	26/261 (9.96%)	0.894 (0.503–1.587)	0.701
- cardioembolism †	497	17/236 (7.20%)	3/261 (1.15%)	0.150 (0.043–0.518)	0.003
- small-vessel occlusion †	497	60/236 (25.42%)	137/261 (52.49%)	3.241 (2.215–4.742)	< 0.001
- other determined etiology †	497	130/236 (55.08%)	93/261 (35.63%)	0.451 (0.315–0.647)	< 0.001
- undetermined etiology †	497	3/236 (1.127%)	2/261 (0.77%)	0.600 (0.099–3.621)	0.577
Side of stroke					
- right hemisphere †	575	95/278 (34.17%)	128/297 (43.10%)	1.459 (1.041–2.045)	0.028
- left hemisphere †	575	134/278 (48.20%)	146/297 (49.16%)	1.039 (0.749–1.441)	0.819
- posterior part †	575	43/278 (15.47%)	16/297 (5.39%)	0.311 (0.171–0.567)	< 0.001
- more than one localization †	575	6/278 (2.16%)	7/297 (2.36%)	1.094 (0.363–3.297)	0.873
rt-Pa treatment †	575	65/278 (23.38%)	80/297 (26.94%)	1.208 (0.828–1.763)	0.327
Thrombectomy †	575	17/278 (6.12%)	7/297 (2.36%)	0.371 (0.151–0.908)	0.030
Medical history					
- hypertension †	572	186/278 (66.91%)	221/294 (75.17%)	1.497 (1.041–2.155)	0.030
- diabetes †	572	70/278 (25.18%)	94/294 (31.97%)	1.397 (0.969–2.012)	0.073
- atrial fibrillation †	573	33/278 (11.87%)	116/295 (39.32%)	4.811 (3.123–7.411)	< 0.001
- myocardial infraction †	572	33/278 (11.87%)	48/294 (16.33%)	1.449 (0.899–2.335)	0.128
- PCI or CABG †	572	20/278 (7.19%)	28/294 (9.52%)	1.358 (0.746–2.471)	0.317
- smoking – ever †	553	138/277 (49.82%)	106/276 (38.41%)	0.628 (0.448–0.881)	0.007
- previous stroke or TIA †	567	48/277 (17.33%)	63/290 (21.72%)	1.324 (0.872–2.011)	0.188
CIRS, total score ‡	572	7 (5–10)	11 (7–14)	1.200 (1.150–1.252)	< 0.001
Aphasia in hospital †	575	74/278 (26.62%)	131/297 (44.11%)	2.176 (1.532–3.090)	< 0.001
Neglect in hospital †	575	21/278 (7.55%)	52/297 (17.51%)	2.597 (1.520–4.440)	< 0.001
Vision deficits in hospital †	575	63/278 (22.66%)	183/297 (61.62%)	5.478 (3.801–7.895)	< 0.001
Delirium in hospital †	575	28/278 (10.07%)	138/297 (46.46%)	7.749 (4.930–12.181)	< 0.001
NIHSS at admission ‡	575	4 (2–8)	13 (5–19)	1.140 (1.110–1.172)	< 0.001
Pre-hospital mRS ‡	574	0 (0–0)	0 (0–3)	2.121 (1.754–2.565)	< 0.001
Pre-hospital IQCODE ‡	475	78 (78–80)	80 (78–90)	1.063 (1.038–1.088)	< 0.001
CRP level in hospital [mg/l] ‡	559	3.43 (1.59–9.78)	13.39 (4.86–43.48)	1.021 (1.014–1.029)	< 0.001

^†^ *n* (%) ‡ median (IQR)

**Table 3:** Significant risk factors of five-year mortality in the univariate and multivariate logistic regression models

	UNIVARIATE REGRESSION MODEL		MULTIVARIATE REGRESSION MODEL ^1^	
VARIABLE	OR (95%CI)	*P*-value	OR (95%CI)	*P*-value
Age (1 year)	1.120 (1.098–1.142)	< 0.001	1.114 (1.079–1.151)	< 0.001
Atrial fibrillation (0/1)	4.811 (3.123–7.411)	< 0.001	2.266 (1.145–4.487)	0.019
Smoking – ever (0/1)	0.628 (0.448–0.881)	0.007	2.817 (1.472–5.391)	0.002
Vision deficits in hospital (0/1)	5.478 (3.801–7.895)	< 0.001	2.968 (1.626–5.416)	< 0.001
Delirium in hospital (0/1)	7.749 (4.930–12.181)	< 0.001	2.887 (1.433–5.813)	0.003
Pre-hospital mRS (1 point)	2.121 (1.754–2.565)	< 0.001	1.525 (1.172–1.984)	0.002
CRP level in hospital (1 mg/l)	1.021 (1.014–1.029)	< 0.001	1.011 (1.003–1.020)	0.006

^1^ adjusted for variables at *p*-value < 0.1 in the univariate analyses, using the forward stepwise selection method

**Fig. 2:**
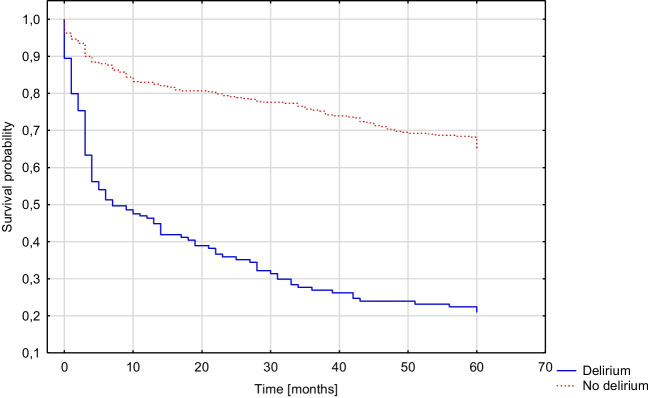
Kaplan–Meier curve presenting the cumulative five-year mortality rate of patients with and without in-hospital post-stroke delirium (Gehan-Wilcoxon test, *p* < 0,001)

In-hospital post-stroke delirium was diagnosed in 203 of 750 participants (27.07%), with 514 examinations based on bCAM and 236 on CAM-ICU. Patients with in-hospital post-stroke delirium had a higher risk for death within five years after stroke (OR 3.361, 95%CI 1.899–5.949, *p* < 0.001) and increased mRS score of ≥ 1 (OR 2.978, 95%CI 1.318–6.728, *p* = 0.009) or ≥ 2 points (OR 2.485, 95%CI 1.365–4.526, *p* = 0.003). These results were significant in the multivariate regression models. There was no significant effect of post-stroke delirium on the frequency of hospital or institution stay (*p* = 0.215). The results are presented in Table 4. Figure 2 illustrates the comparison of survival probability between patients with and without delirium (*p* < 0.001).

**Table 4:** Influence of in-hospital post-stroke delirium on five-year prognosis

		INCIDENCE, *n* (%)		UNIVARIATE REGRESSION MODEL		MULTIVARIATE REGRESSION MODEL ^1^	
VARIABLE	Data	NO DELIRIUM (*n* = 547)	DELIRIUM (*n* = 203)	OR (95%CI)	*P*-value	OR (95%CI)	*P*-value
Mortality	575	159/409 (38.88%)	138/166 (83.13%)	7.749 (4.930–12.181)	< 0.001	3.361 (1.899–5.949)	< 0.001
Increase in mRS of ≥ 1	573	274/407 (67.32%)	158/166 (95.18%)	9.587 (4.574–20.091)	< 0.001	2.978 (1.318–6.728)	0.009
Increase in mRS of ≥ 2	573	216/407 (53.07%)	140/166 (84.34%)	4.761 (3.001–7.555)	< 0.001	2.485 (1.365–4.526)	0.003
Hospital or institution stay	278	7/250 (2.80%)	3/28 (10.71%)	4.166 (1.013–17.127)	0.048	2.785 (0.552–14.063)	0.215

^1^ adjusted for age, gender, years of education, CIRS score and NIHSS score

Dementia was diagnosed in 139 of 449 examined participants (30.96%) three months after stroke. 311 patients underwent a full neuropsychological examination, of which 93 (29.90%) were diagnosed with dementia. A total of 138 patients were contacted by telephone, of whom 46 (33.33%) were diagnosed with dementia – 28 underwent T-MoCA (13 with dementia, 46.43%) and 110 had IQCODE conducted with an informant (33 with dementia, 30.00%). There was no significant difference between the rates of patients with dementia according to the site of assessment (outpatient neuropsychological examination vs. phone interview, *p* = 0.468) or the diagnostic method (full neuropsychological examination vs. T-MoCA vs. IQCODE, *p* = 0.187).

**Fig. 3:**
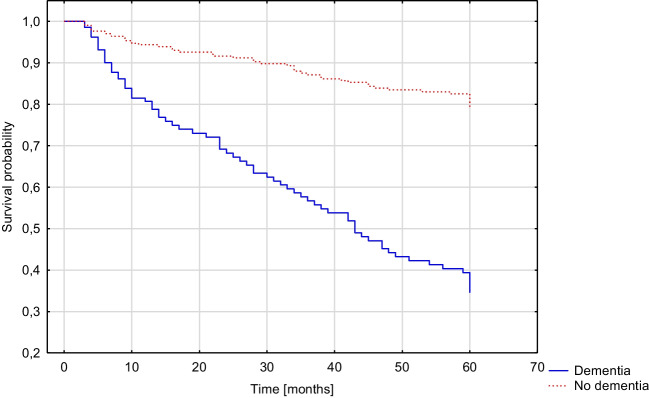
Kaplan–Meier curve presenting the cumulative five-year mortality rate of patients with and without dementia diagnosed three months after stroke (Gehan-Wilcoxon test, *p* < 0,001)

Patients with dementia diagnosed three months after stroke had a higher risk for death within five years following stroke (OR 3.175, 95%CI 1.699–5.934, *p* < 0.001) and increased mRS score of ≥ 1 (OR 3.561, 95%CI 1.606–7.895, *p* = 0.002) or ≥ 2 points (OR 3.344, 95%CI 1.679–6.659, *p* = 0.001). These results were significant in the multivariate regression models. Adjusting for the presence of in-hospital delirium had no noticeably effect on the results. There was no significant effect of post-stroke dementia on the frequency of hospital or institution stay (*p* = 0.603). The results are presented in Table 5. Figure 3 compares the survival probability of patients with and without dementia diagnosed three months after stroke (*p* < 0.001).

**Table 5:** Influence of dementia diagnosed three months after stroke on five-year prognosis

		INCIDENCE, *n* (%)		UNIVARIATE REGRESSION MODEL		MULTIVARIATE REGRESSION MODEL ^1^		MULTIVARIATE REGRESSION MODEL ^2^	
VARIABLE	Data	NO DEMENTIA (*n* = 310)	DEMENTIA (*n* = 139)	OR (95%CI)	*P*-value	OR (95%CI)	*P*-value	OR (95%CI)	*P*-value
Mortality	333	51/224 (22.77%)	73/109 (66.97%)	6.879 (4.144–11.418)	< 0.001	3.175 (1.699–5.934)	< 0.001	2.831 (1.490–5.379)	0.001
Increase in mRS of ≥ 1	331	131/223 (58.74%)	99/108 (91.67%)	7.725 (3.713–16.072)	< 0.001	3.561 (1.606–7.895)	0.002	3.296 (1.472–7.377)	0.004
Increase in mRS of ≥ 2	331	97/223 (43.50%)	92/108 (85.19%)	7.469 (4.127–13.518)	< 0.001	3.344 (1.679–6.659)	0.001	2.949 (1.463–5.944)	0.002
Hospital or institution stay	209	4/173 (2.31%)	3/36 (8.33%)	3.841 (0.821–17.966)	0.087	1.743 0.215–14.122)	0.603	1.168 (0.106–12.808)	0.899

^1^ adjusted for age, gender, years of education, CIRS score and NIHSS score

^2^ adjusted for age, gender, years of education, CIRS score, NIHSS score and incidence of in-hospital post-stroke delirium

Twelve months after the stoke, 171 of 477 participants (35.85%) were diagnosed with dementia. 256 patients underwent a full neuropsychological examination, of which 87 (33.98%) were diagnosed with dementia. 221 patients were contacted by phone, of whom 84 (38.01%) were diagnosed with dementia – 79 underwent T-MoCA (42 with dementia, 53.16%) and 142 had IQCODE conducted with an informant (42 with dementia, 29.58%). There was no significant difference between the rates of demented patients according to the site of assessment (outpatient neuropsychological examination vs. phone interview, *p* = 0.361), but dementia was significantly more often diagnosed with T-MoCA compared to a full neuropsychological examination and IQCODE (*p* = 0.001).

**Fig. 4:**
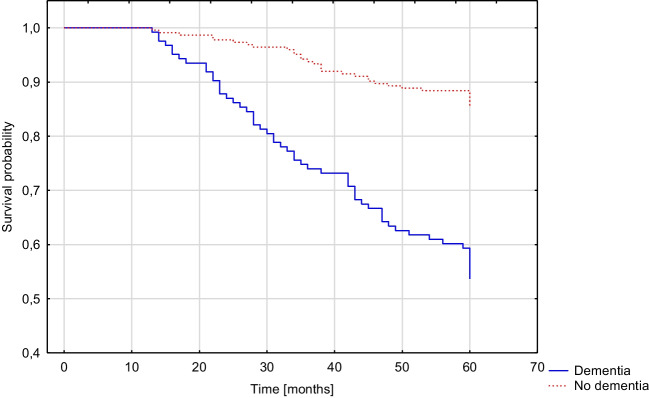
Kaplan–Meier curve presenting the cumulative five-year mortality rate of patients with and without dementia diagnosed twelve months after stroke (Gehan-Wilcoxon test, *p* < 0,001)

Patients with dementia diagnosed twelve months after stroke had a higher risk for death within five years following stroke (OR 1.917, 95%CI 1.014–3.623, *p* = 0.045) and increased mRS score of ≥ 1 (OR 2.213, 95%CI 1.175–4.168, *p* = 0.014) or ≥ 2 points (OR 2.148, 95%CI 1.187–3.886, *p* = 0.012). These results were significant in the multivariate regression models. After adjusting for the presence of in-hospital delirium, post-stroke dementia was not an independent risk factor for mortality (*p* = 0.111), but the effect on disability based on change in mRS score remained significant. There was no significant effect of post-stroke dementia on the frequency of hospital or institution stay (*p* = 0.052). The results are shown in Table 6. Figure 4 compares the survival probability of patients with and without dementia diagnosed twelve months after stroke (*p* < 0.001).

**Table 6:** Influence of dementia diagnosed twelve months after stroke on five-year prognosis

		INCIDENCE, *n* (%)		UNIVARIATE REGRESSION MODEL		MULTIVARIATE REGRESSION MODEL ^1^		MULTIVARIATE REGRESSION MODEL ^2^	
VARIABLE	Data	NO DEMENTIA (*n* = 306)	DEMENTIA (*n* = 171)	OR (95%CI)	*P*-value	OR (95%CI)	*P*-value	OR (95%CI)	*P*-value
Mortality	347	33/224 (14.73%)	57/123 (46.34%)	4.999 (2.996–8.341)	< 0.001	1.917 (1.014–3.623)	0.045	1.699 (0.885–3.263)	0.111
Increase in mRS of ≥ 1	345	112/223 (50.22%)	102/122 (83.61%)	5.054 (2.926–8.730)	< 0.001	2.213 (1.175–4.168)	0.014	2.102 (1.112–3.971)	0.022
Increase in mRS of ≥ 2	345	74/223 (33.18%)	89/122 (72.95)	5.430 (3.336–8.838)	< 0.001	2.148 (1.187–3.886)	0.012	2.009 (1.102–3.661)	0.023
Hospital or institution stay	257	2/191 (1.05%)	8/66 (12.12%)	13.034 (2.692–63.105)	0.001	6.421 (0.987–41.759)	0.052	6.103 (0.917–40.604)	0.061

^1^ adjusted for age, gender, years of education, CIRS score and NIHSS score

^2^ adjusted for age, gender, years of education, CIRS score, NIHSS score and incidence of in-hospital post-stroke delirium

Table 7 compares five-year outcomes between ischemic stroke, hemorrhagic stroke and TIA. Patients after TIA were significantly less likely to die five years after stroke (*p* = 0.003), and their mRS score was significantly less likely to increase by ≥ 1 (*p* = 0.005) or ≥ 2 points (*p* = 0.009). There was no difference in the frequency of hospital or institution stay (*p* = 0.901).

**Table 7:** Comparison of the five-year outcomes between patients with ischemic stroke, hemorrhagic stroke and TIA

VARIABLE	Data	ISCHEMIC STROKE (*n* = 650)	HEMORRHAGIC STROKE (*n* = 52)	TIA (*n* = 48)	*P*-value
Mortality	575	263/498 (52.81%)	26/44 (59.09%)	8/33 (24.04%)	0.003
Increase in mRS of ≥ 1	573	377/497 (75.86%)	38/44 (86.36%)	17/32 (53.13%)	0.005
Increase in mRS of ≥ 2	573	313/497 (62.98%)	31/44 (70.45%)	12/32 (37.50%)	0.009
Hospital or institution stay	278	8/235 (3.40%)	1/18 (5.56%)	1/25 (4.00%)	0.901

## Discussion

The study analyzed the five-year prognosis of patients after an acute stroke or TIA in the Polish population. More than half of the observed participants died during this time. Sociodemographic and stroke-related factors affecting five-year mortality were identified. Post-stroke delirium and post-stroke dementia are independent risk factors for mortality and disability, but there is no significant association with institutionalization rates.

The five-year post-stroke mortality rate was 51.65%, which is comparable to previous studies in which the death rate at the same time point ranged from 35 to 71% [28–31], but data from such a long follow-up after stroke are still limited. We realize that these results may vary depending on the methodology, the population studied, the country and healthcare system, sociodemographic factors and the effectiveness of follow-up reporting. We also anticipate that the mortality rate may be underestimated or overestimated due to the loss of almost a quarter of patients during follow-up. Patients lost during follow-up differed from the included participants in factors that proved to be predictors of mortality in the further analysis. They were less likely to suffer from atrial fibrillation, were less likely to present vision deficits and delirium in hospital, and had lower pre-hospital mRS scores, which may suggest that patients lost to follow-up were more likely to survive than those who were included in the study. On the other hand, the inability to contact some participants appears to be associated with a high probability of death per se.

Several factors such as older age, atrial fibrillation, pre-stroke smoking, in-hospital visual deficits, in-hospital delirium, pre-hospital mRS and higher in-hospital CRP levels, were found to be independent predictors of mortality five years after stroke. These factors are also reported in the literature by other authors, but the points of observation are different [28, 32–35].

In our study, in-hospital post-stroke delirium more than tripled the risk of death and was an independent risk factor of disability five years after stroke, defined as an increase in mRS score. Delirium has previously been shown to increase the risk of death at five-year follow-up among post-operative patients [36], but there is a lack of studies on post-stroke patients in this perspective. Shi et al. in 2012 summarized in their meta-analysis that post-stroke delirium was associated with higher one-year mortality [37], and these results have also been confirmed by subsequent authors [38, 39]. Although the negative impact of post-stroke delirium on functional disability is known [38, 40], long-term data were still lacking.

We found that post-stroke dementia was an independent risk factor of five-year mortality and disability. Because the most appropriate time for follow-up neuropsychological testing among stroke survivors is ambiguous [9], we conducted two analyses based on cognitive assessment three and twelve months after stroke. Compared to the literature, Barba et al. showed that post-stroke dementia was a strong independent risk factor for early death, with a mortality rate up to 8.5 higher compared to patients without dementia [41], Harnod et al. showed increased mortality in patients with post-stroke dementia at twelve-year follow-up [42], while Cumming et al. found an association between post-stroke dementia and lower quality of life at one-year follow-up [43].

Recovery from stroke can take many years [44, 45]. Therefore, we strongly believe that our long-term observations will be incorporated into clinical practice to initiate appropriate management and to prevent specific complications of stroke. Knowledge of the worse prognosis of patients with delirium and dementia after stroke may allow clinicians to place special emphasis on identifying high-risk cases of these complications.

In the present study, we did not observe an effect of post-stroke delirium and post-stroke dementia on the rate of institutionalization including rehabilitation hospital or long-term facilities such as nursing homes. The overall five-year institutionalization rate in our stroke population was lower (3.60%) than in the literature, where it ranged from 11 to 19% [46]. Although the number of patients placed in institutions was higher in the delirium and dementia groups, it is difficult to prove the significance of the difference between two such low event rates.

Trends in the institutionalization of patients with disabilities after hospital discharge vary from country to country and society to society due to different healthcare systems, social conditions and customs [47]. To date, there are no reliable data on this subject in the Polish population and among post-stroke patients. However, based on our experience, we see a tendency among Polish families to care for their disabled relatives after stroke at home. In addition, the frequency of institutionalization was also lower compared to our earlier observation on prognosis at twelve months after stroke [34]. We expect that severely disabled patients who required more intensive and advanced care may have mostly died by the time point of five-year follow-up.

Our study has several methodological strengths and limitations. The advantages include large number of patients analyzed, prospective design, complex evaluation of the neuropsychiatric status and the very long follow-up time. This study is a continuation of the “PROPOLIS” project [12] and newly collected data five years after stroke offers the prospect of other analyses of neuropsychologic status and prognosis among stroke patients at different time points including at hospital admission, as well as at follow-up points at three months, twelve months and five years after stroke.

The weakness of our study included, in turn, a relatively high percentage of patients lost to follow-up, which may have affected the results. In addition, we analyzed adult patients in a wide age range, but in the end the inclusion of young patients did not significantly affected the mean age of our study population compared to other studies. Most of the participants in our study did not receive regular neurological care after discharge at our center, so our cohort may have differed in terms of the level of post-stroke rehabilitation plan, secondary prevention, and treatment of stroke complications. Finally, different diagnostic methods, which have different sensitivity and specificity in diagnosing dementia, were used three and twelve months after stroke. The T-MoCA may result in overdiagnosis of dementia compared to other methods.

## Conclusions

More than half of post-stroke patients die during the five-year follow-up. Post-stroke delirium and post-stroke dementia negatively affect the long-term prognosis, being associated with an increased risk of death and disability.

## Data Availability

The datasets generated during and/or analysed during the current study are available from the corresponding author on reasonable request.
